# *dxpr*: an R package for generating analysis-ready data from electronic health records—diagnoses and procedures

**DOI:** 10.7717/peerj-cs.520

**Published:** 2021-05-26

**Authors:** Yi-Ju Tseng, Hsiang-Ju Chiu, Chun Ju Chen

**Affiliations:** 1 Department of Information Management, National Central University, Taoyuan, Taiwan; 2 Department of Laboratory Medicine, Chang Gung Memorial Hospital at Linkou, Taoyuan, Taiwan; 3 Department of Information Management, Chang Gung University, Taoyuan, Taiwan; 4 Department of Information Management, National Taiwan University, Taipei, Taiwan

**Keywords:** Electronic health records, Analysis-ready data, Exploratory data analysis, R package

## Abstract

**Background:**

Enriched electronic health records (EHRs) contain crucial information related to disease progression, and this information can help with decision-making in the health care field. Data analytics in health care is deemed as one of the essential processes that help accelerate the progress of clinical research. However, processing and analyzing EHR data are common bottlenecks in health care data analytics.

**Methods:**

The *dxpr* R package provides mechanisms for integration, wrangling, and visualization of clinical data, including diagnosis and procedure records. First, the *dxpr* package helps users transform International Classification of Diseases (ICD) codes to a uniform format. After code format transformation, the *dxpr* package supports four strategies for grouping clinical diagnostic data. For clinical procedure data, two grouping methods can be chosen. After EHRs are integrated, users can employ a set of flexible built-in querying functions for dividing data into case and control groups by using specified criteria and splitting the data into before and after an event based on the record date. Subsequently, the structure of integrated long data can be converted into wide, analysis-ready data that are suitable for statistical analysis and visualization.

**Results:**

We conducted comorbidity data processes based on a cohort of newborns from Medical Information Mart for Intensive Care-III (n = 7,833) by using the *dxpr* package. We first defined patent ductus arteriosus (PDA) cases as patients who had at least one PDA diagnosis (ICD, Ninth Revision, Clinical Modification [ICD-9-CM] 7470*). Controls were defined as patients who never had PDA diagnosis. In total, 381 and 7,452 patients with and without PDA, respectively, were included in our study population. Then, we grouped the diagnoses into defined comorbidities. Finally, we observed a statistically significant difference in 8 of the 16 comorbidities among patients with and without PDA, including fluid and electrolyte disorders, valvular disease, and others.

**Conclusions:**

This *dxpr* package helps clinical data analysts address the common bottleneck caused by clinical data characteristics such as heterogeneity and sparseness.

## Introduction

On the basis of the development of electronic health records (EHRs), data analytics in health care is deemed as an essential process for accelerating the progress of clinical research (Hersh, 2007; Jensen, Jensen & Brunak, 2012; Miotto & Weng, 2015). Enriched EHRs contain crucial information related to disease progression, and this information can help with decision making in the health care field including for treatment selection and disease diagnosis (Jensen, Jensen & Brunak, 2012; Raghupathi & Raghupathi, 2014). However, processing and analyzing EHR data are usually challenging because of their heterogeneity and sparsity. These inherent characteristics create a common bottleneck in health care big data analytics (Wu, Roy & Stewart, 2010; Hripcsak & Albers, 2013; Weiskopf & Weng, 2013). Moreover, executing clinical data analysis project across different departments or institutes is difficult because clinical data formats and terminologies used to describe clinical conditions may vary across departments. A method that can standardize and facilitate the sharing of data or analysis pipelines from multiple sources is needed in research on clinical data analysis. Several common data models (CDMs) have been developed for eliminating clinical data format barriers, including the National Patient-Centered Clinical Research Network (PCORnet) (Fleurence et al., 2014; PCORnet, 2020) and Observational Medical Outcomes Partnership (OMOP) CDM (Observational Health Data Sciences and Informatics, 2020). The concept of CDM is to transform data into a CDM and terminology and then allow users to perform systematic analyses by using various sources. Although a CDM can help perform systematic analyses across different sources, the integration of clinical data and the preparation of analysis-ready data are unsolved issues.

The proposed open-source *dxpr* R package is a software tool aimed at expediting general EHR or claims data analyses through incorporating several functions that enable users to standardize, integrate, wrangle, and visualize clinical diagnosis and procedure records. Preparing an analysis-ready dataset from EHRs or claims data is a complex task that requires both medical knowledge and data science skills. The proposed *dxpr* package simplifies and accelerates the workflow for EHR data extraction and helps clinical data analysts generate simple and clean scripts that can easily be shared and reproduced. The *dxpr* package enables researchers to explore EHRs or claims data to acquire crucial information, understand disease progression, and analyze outcomes without writing complicated data preprocessing scripts. Moreover, the proposed package can support collaborative research across multiple data sources as long as the data include general diagnosis- or procedure-related information.

**Figure 1:**
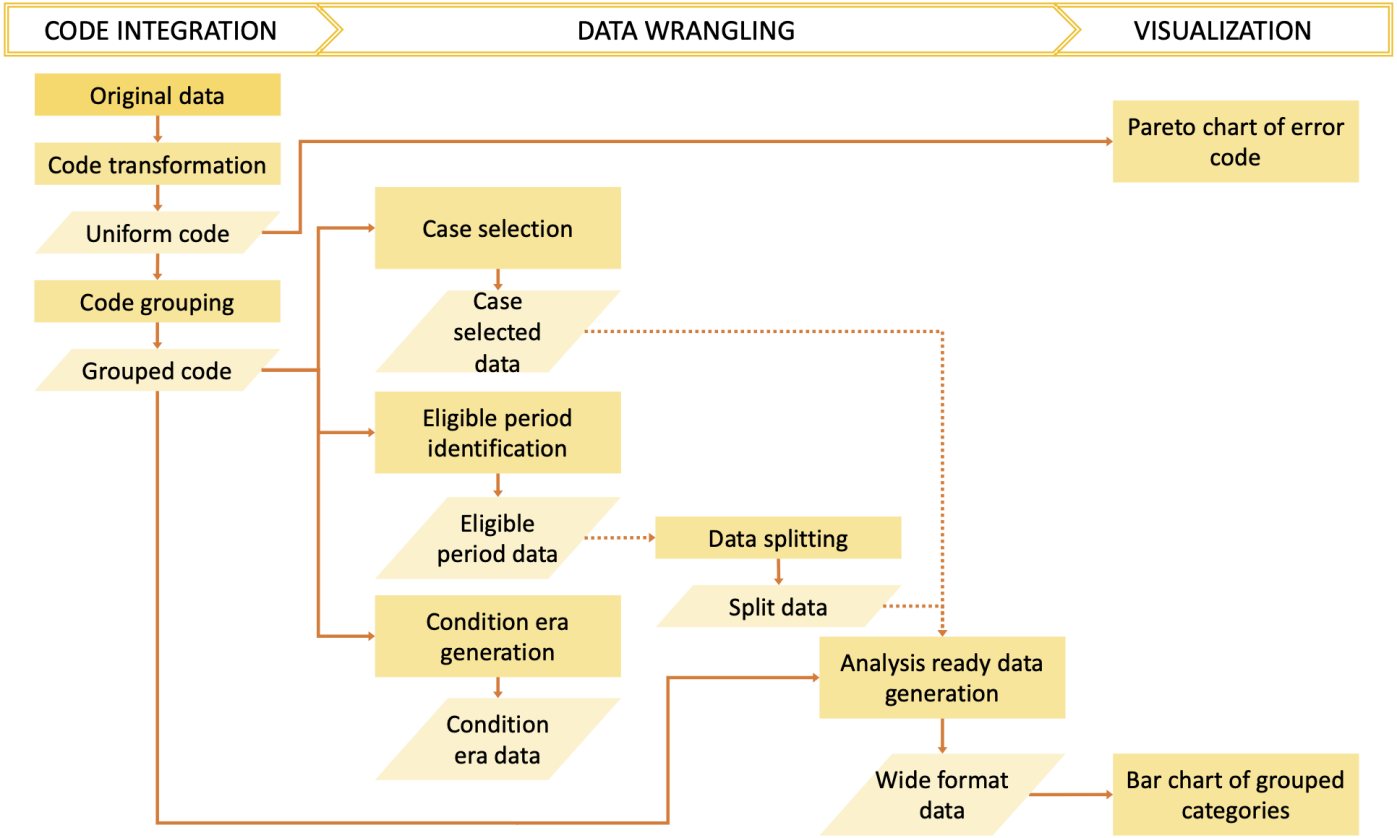
Overview of the *dxpr* package.

The *dxpr* package has three phases to process and analyze diagnosis codes in EHRs (Fig. 1). In the first phase, namely data integration, we transform diagnosis codes into a uniform format and provide four strategies to group diagnoses into clinically meaningful categories before the wrangling process. In the second phase, namely, data wrangling, users can use provided functions to query eligible cases, split data based on the index date, and calculate condition era according to the grouped diagnostic categories of each patients. Furthermore, exploratory data analysis preparation can be performed in this phase. Moreover, the *dxpr* package provides a function to convert a long format of grouped data into a wide format, which fits other analytical and plotting functions from other packages better. In the last phase, namely visualization, we provide overviews for diagnosis standardization and data integration, such as comorbidity distribution in the study population, comorbidity differences between case and control groups, and the most common diagnoses that failed to be grouped or transformed. The usage details are presented in the Supplementary Data S1 and S2. For processing and analyzing procedure codes, the concept is similar to diagnosis.

## Materials and Methods

### Preparation

The current version of the package is available at Github (https://github.com/DHLab-TSENG/dxpr, Supplementary Data S3) and is accessible through the devtools package that enables installing packages from GitHub (Wickham, Hester & Chang, 2020). To install the *dxpr* R package, users can type the following commands in an R session:


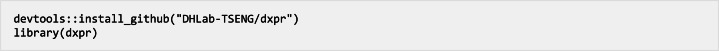


The imported EHR dataset must contain at least three columns as indicated below:

• Member ID: a patient identifier, which can be numeric, alphanumeric, or a list of characters.
• Diagnosis/procedure code: ICD-9 or ICD-10 code assigned to a visit or an admission.
• Visit or admission date: the date of the visit, admission, or clinical service provided. The date should be in date format. If the date is recorded in a string format, it has to be recorded in year–month–day format (YYYY/MM/DD or YYYY-MM-DD).

Column names can be passed in each function by using function arguments.

The data can be imported from files or databases, with packages provide access to databases within R, such as DBI (R Special Interest Group on Databases R-SIG-DB, Wickham & Müller, 2021) and odbc (Hester & Wickham, 2021). We illustrate the use of the *dxpr* package with a diagnostic sample dataset of 10-year admissions of 38 patients, sampleDxFile, and the first five records are shown in Table 1.

**Table 1:** The first five diagnosis records of the sample dataset.

**ID**	**ICD**	**Date**
A2	Z992	2020-05-22
A5	Z992	2020-01-24
A8	Z992	2015-10-27
A13	Z992	2020-04-26
A13	Z992	2025-02-02

### Data integration

#### Code format transformation

The *dxpr* package first transforms ICD diagnostic codes into a uniform format before code grouping. ICD-9 and ICD-10 diagnostic codes (US Centers for Medicare & Medicaid Services, 2017a) have two formats, namely decimal (with a decimal place separating the code) and short formats. Different hospitals, grouping methods, or standards coded ICD into different formats. For example, studies using Clinical Classifications Software (CCS) (HCUP, 2017; HCUP, 2019a) and comorbidity measures, such as Elixhauser and Charlson (Elixhauser et al., 1998; Menendez et al., 2014; Moore et al., 2017), have coded the ICD in a short format, and a phenome-wide association study (PheWAS) (Denny et al., 2010) coded the ICD in a decimal format. Therefore, format transformation is required before code grouping, and the transformation type is decided by the chosen grouping method.

The transformation function (icdDxShortToDecimal) converts ICD-9 and ICD-10 codes into a uniform decimal format because a decimal format is needed for grouping diagnostic codes in PheWAS classification. Similar to icdDxShortToDecimal, icdDxDecimalToShort function converts diagnostic codes into a uniform short format, which can be used for grouping to CCS, Elixhauser, or other classifications. These transformative functions not only convert ICD codes into uniform format codes but also check for potential coding errors. We provide two types of warning messages: wrong ICD format and wrong ICD version. Additional suggestions are generated to help users adjust potential incorrect ICD codes if available.


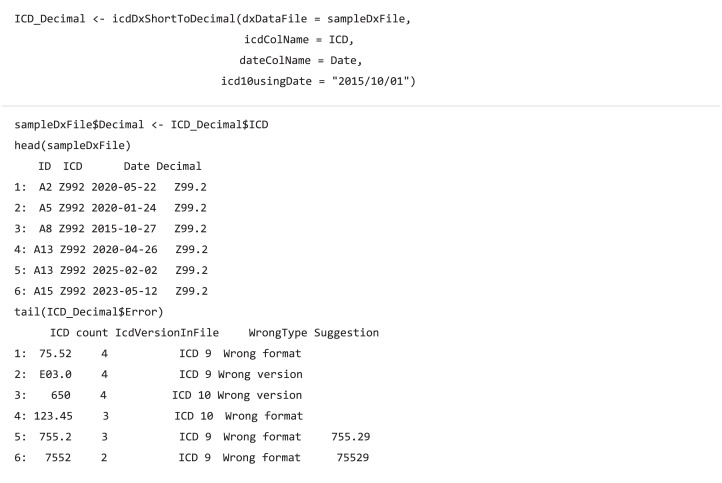


#### Code grouping

The code grouping functions collapse clinical diagnostic data (ICD-9/ICD-10 codes) (US Centers for Medicare & Medicaid Services, 2017a) into a smaller number of clinically meaningful categories that are more useful for presenting descriptive statistics than using individual diagnostic codes (HCUP, 2019b). The *dxpr* package supports four strategies to group EHR diagnosis codes, namely CCS (US Centers for Medicare & Medicaid Services, 2017b), PheWAS (Denny et al., 2010) (icdDxToPheWAS), comorbidity measures (Elixhauser et al., 1998; Menendez et al., 2014; Moore et al., 2017), and self-defining grouping methods. The CCS grouping strategies includes single-level CCS (icdDxToCCS) and multiple-level CCS (icdDxToCCSLvl) (HCUP, 2017; HCUP, 2019a), comorbidity measures (icdDxToComorbid) includes Elixhauser, Agency for Healthcare Research and Quality (AHRQ) and Charlson (Elixhauser et al., 1998; Menendez et al., 2014; Moore et al., 2017), and self-defining grouping methods includes precise matching (icdDxToCustom) and searching for lines containing a match (icdDxToCustomGrep). The grouping functions return two tables of the dataset, one is data with the corresponding grouping categories of each ICD (Table 2), and the other is summarized data exhibiting the earliest/latest record date and diagnosis counts in the same grouping category for each patient (Table 3). For example, after executing function icdDxToCCS for the records of patients A and B, two output types are shown in Tables 2 and 3, respectively. Patient A has three diagnosis records (ICD codes: 78550, 78552, and 785.59), which are all in the “shock” category of the CCS classification, with the earliest record on September 1, 2013 and the latest one on October 1, 2014. The icdDxToCCS function mapped corresponding CCS categories for these ICD codes and returned the grouping results (Table 2). Similarly, patient B has two diagnosis records (ICD codes: 78552 and 250.00) in the “shock” category and “Diabetes mellitus without complication” category of CCS classification, and the grouping results are also shown in Table 2. According to these diagnosis records shown in Table 2, Table 3 shows that icdDxToCCS function can summarize the first and last dates of diagnosis, the total number of diagnoses, and the period between the first and last diagnoses for each category, which can be used for designing the analysis strategy. While icdDxToCCS groups codes into single-level CCS, icdDxToCCSLvl groups codes into multi-level CCS. Multi-level CCS expands single-level CCS into a four-level hierarchical system for diagnoses, which provide the opportunity to examine general aggregations or to assess specific conditions (HCUP, 2019a). For instance, if a user wishes to group codes into the second level of multi-level CCS, then this task can be performed through simply entering “ccslvl2” as the assigned grouping type. These grouping functions not only facilitate users to convert original diagnosis records from detailed levels into clinically meaningful diagnostic groups for further analysis but also provide aggregated information of each diagnostic group that can help research design and hypothesis generation, such as filtering out data based on specified criteria (e.g., first diagnosis dates of a specific chronic disease).

**Table 2:** Grouping results from grouping functions—icdDxToCCS.

Short	**ID**	**ICD**	**Date**	ccs_categories_description
78550	A	78550	2014/10/01	Shock
78552	A	78552	2013/10/01	Shock
78559	A	785.59	2013/09/01	Shock
78552	B	78552	2013/09/01	Shock
25000	B	250.00	2012/07/01	Diabetes mellitus without complication
25000	B	250.00	2012/05/01	Diabetes mellitus without complication

**Table 3:** Summarized results from grouping functions—icdDxToCCS.

ID	Categories	FirstCaseDate	EndCaseDate	Count	Period
A	Shock	2013/09/01	2014/10/01	3	395 days
B	Diabetes mellitus without complication	2012/05/01	2012/07/01	2	62 days
B	Shock	2013/09/01	2013/09/01	1	0 days

The usage of code classification function for CCS is as follows:


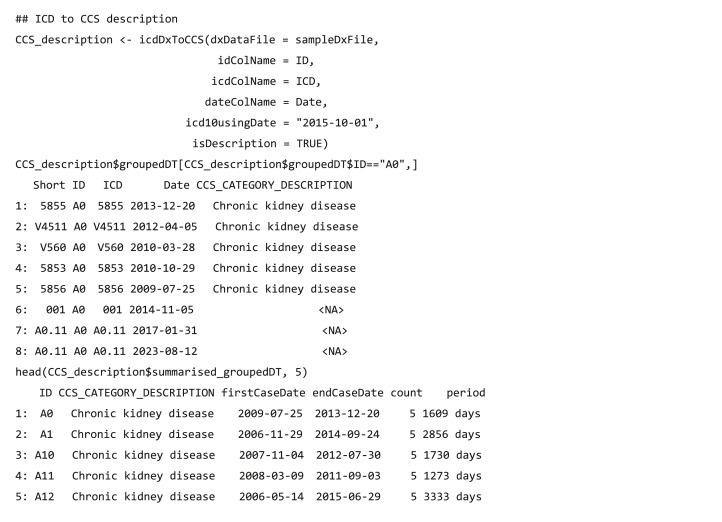


### Data wrangling

#### Case selection

In clinical data analysis projects, the most crucial step is case definition and selection, such as defining Lyme disease cases from claims data (Tseng et al., 2015) or defining acute ischemic stroke from EHR (Tseng et al., 2020). The analysis results could change based on case definition and lead to a different conclusion. The query function selectCases can select cases matching case definitions. Users can select cases based on diagnosis (ICD) or diagnostic categories (CCS, PheWAS, comorbidities, or self-defined diagnostic categories). Moreover, the function provides an option to set the minimum number of diagnoses within a specific duration. For example, users can extract diabetes cases by assigning at least two diagnoses in ICD codes “250.xx” or “E10.x-E14.x” within 730 days when a user applies the validated diabetes case definition: “two physician claims within 2 years with diagnosis codes 250.xx or E10.x-E14.x” (Chen et al., 2010). The output dataset of this function provides the start and end dates of the cases, the number of days between them, and the most common ICD codes used in the case definition. Furthermore, a list of people who did not satisfy the required case conditions or practically match the case definition is appended in the returned output table, and these individuals can be defined as a control group or be removed.


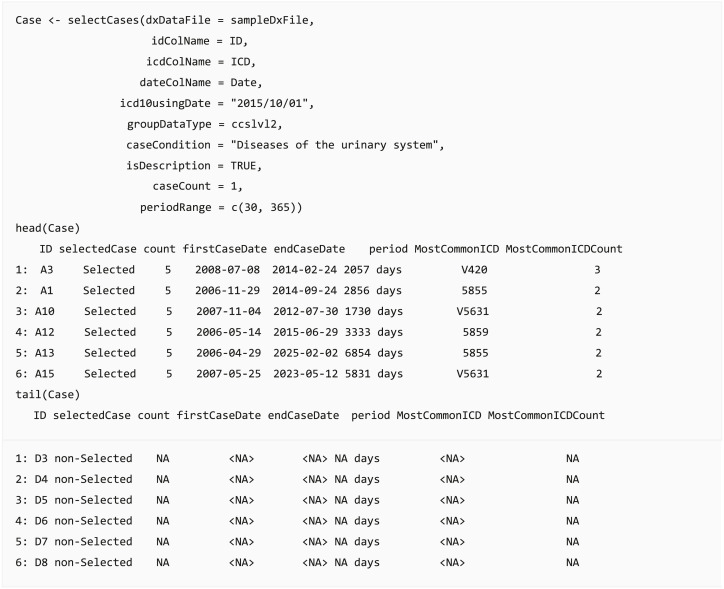


#### Eligible period identification

In some clinical data, such as claims data, individuals can join or leave the program on different dates, and the length of available records might affect the analysis completeness. The *dxpr* package provides a function getEligiblePeriod for researchers to identify the first/last record date for each patient. These outputs can be used as an index date for case exclusion, such as cases without at least 6 months washout or follow-up period, or further data splitting.


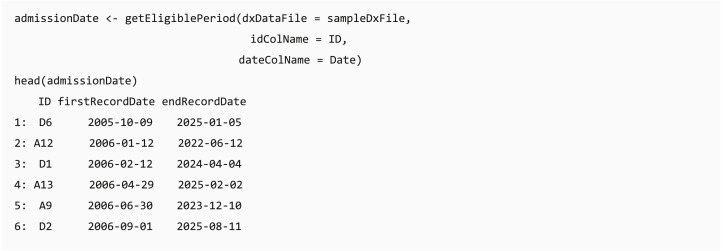


#### Data splitting based on index date and moving window

In clinical data analysis projects, users usually need to extract data based on a specific clinical event (e.g., extracting data before the first Lyme disease diagnosis in the records (Tseng et al., 2017)). The date of the specific event (index date) can be the first/last record date of the events or patient record, and the table of the index date for each individual can be generated using selectCases or getEligiblePeriod function, respectively. The *dxpr* package provides a convenient function splitDataByDate that can split data through classifying the data recorded before or after the defined index date and calculating the period between the record date and index date based on a self-defined window. For example, if a user needs to aggregate the data by using a 30-day window, the data recorded on 15 and 45 days after the index date will be defined as window 1 and window 2, respectively. The output of splitDataByDate function helps users to split the data based on the study design, and this can be applied to further time-series multiple-measurement analysis with period information.


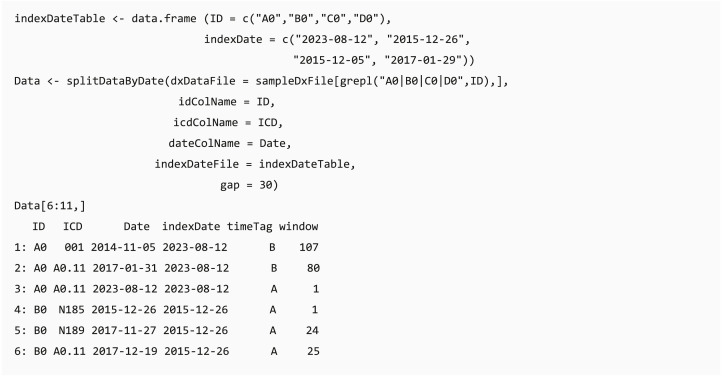


#### Condition era generation

Condition era is a means to apply consistent rules for medical conditions to infer distinct episodes in care, generated through integrating distributed clinical records into a single progression record (Reisinger et al., 2010). The concept of condition era is committed to the length of the persistence gap: when the time interval of any two consecutive admissions for certain conditions is smaller than the length of the persistence gap, then these two admission events will be aggregated into the same condition era. Each condition era consists of one or many events, and differences between any two consecutive admission events are all within the persistence gap. For example, an episode of influenza may include single or multiple outpatient visits, and the length of the influenza course should be the period between the first and last visits of the episode. getConditionEra function calculates condition era by using the grouped categories or self-defining groups of each patient and then generates a table with individual IDs, the first and last record of an era, and the sequence number of each episode. Users can easily convert scattered diagnoses into an episode of condition based on the chararistics of target disease progression with the proposed function.


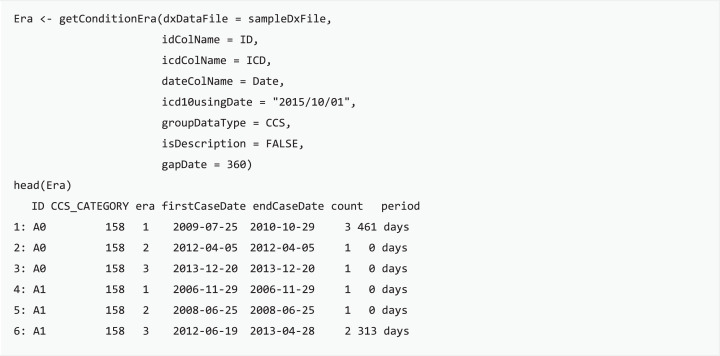


#### Analysis-ready data generation

After data integration and wrangling, researchers often need to further analyze these processed data, and function groupedDataLongToWide converts the long format of grouped data into a wide format, which is fit for other analytical and plotting packages, such as tableone (Yoshida & Bartel, 2020) package.


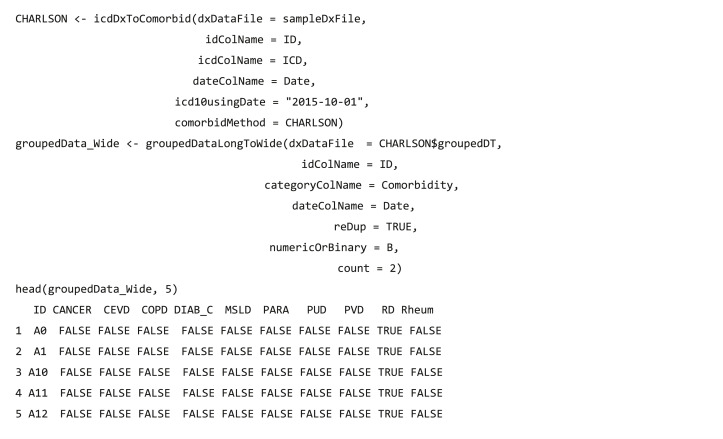


### Visualization

#### Pareto chart of error ICD

When code transformation is implemented in the *dxpr* package, it generates unified data of diagnosis codes with potential errors. Function plotICDError visualizes codes with potential error by using the Pareto chart containing a bar plot where error ICD codes are arranged in descending order, and the cumulative total is represented by the line. Users can sort based on the counts of error ICD codes and set the top selected number of the ordered dataset. For instance, if a user chooses the top 10 ordinal rankings, then the Pareto chart shows a plot of the top 10 common error ICD codes and a list with details of these 10 and other error ICD codes.


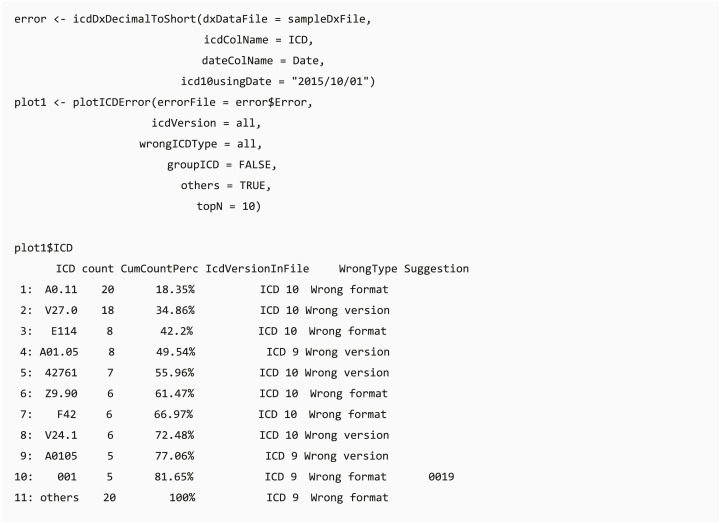


#### Bar chart of diagnostic categories

Function plotDiagCat provides an overview of the grouping categories of the diagnoses and summarizes the proportion of individuals diagnosed with grouped diagnostic categories in the whole study population or case and control groups in a bar chart. Users can observe the number and percentage of diagnostic categories in their dataset through this function. Furthermore, this function compares the usage of significantly different diagnostic categories between case and control groups by using the chi-square test or Fisher’s exact test when the data does not match the assumptions of the chi-square test. The default level of statistical significance is considered at 5% (*p* = 0.05). Researchers can set a threshold of the top N significant grouped categories and the minimum prevalence of the diagnostic groups in the case or control group.

The “percentage” column shows the proportion of individuals diagnosed with the diagnostic category in the group. For example, there are 38 patients in the sample file, and “Renal Failure” defined in Elixhauser comorbidity accounts for 63.16% of the population (24/38).


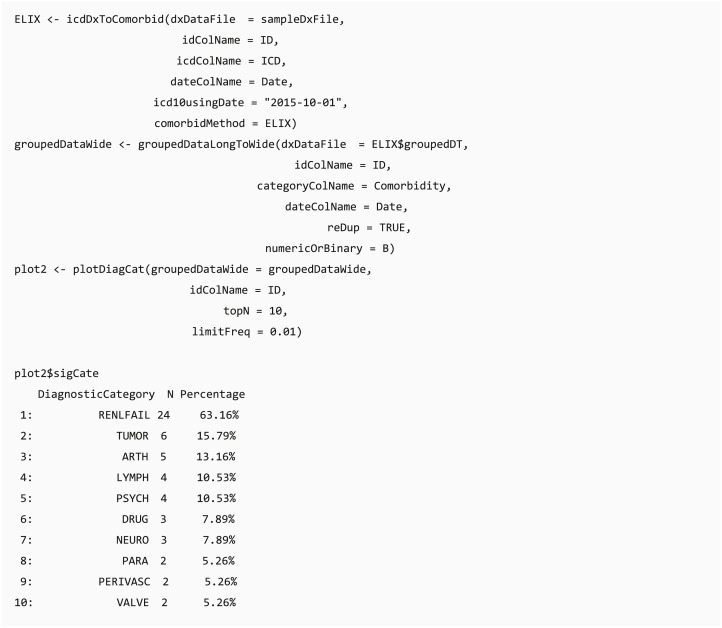


### Clinical procedure data processing

As diagnosis codes, ICD-9-Procedure Coding System (PCS) code also has two formats, namely decimal and short, whereas ICD-10-PCS code only has a short format. The functions (icdPrToCCS and icdPrToProcedureClass) provide two strategies (CCS and procedure class) to collapse ICD procedure codes into clinically meaningful categories for further analysis. This procedure has two CCS classifications: single and multiple levels. The usage is similar to the diagnostic CCS classification. A sample file (samplePrFile) is provided with procedure records, including three patients and 170 records.

The procedure classes (HCUP, 2016) are created to facilitate health services research on hospital procedures by using administrative data. The procedure classes provide a standard to categorize individual procedure codes into one of the four broad categories: minor diagnostic, minor therapeutic, major diagnostic, and major therapeutic. The aforementioned classification functions mentioned allow the researcher to readily determine whether a procedure is diagnostic or therapeutic and whether a procedure is minor or major in terms of invasiveness, resource use, or both.

### Use case

To illustrate the main features in the *dxpr* package and the typical workflow, we demonstrated an analysis using the package among newborns who were diagnosed with patent ductus arteriosus (PDA) from Medical Information Mart for Intensive Care-III (MIMIC-III) (Johnson et al., 2016). MMIC-III is a publicly available database comprising deidentified health-related data associated with the admissions of approximately 60,000 patients who stayed in the critical care units of the Beth Israel Deaconess Medical Center between 2001 and 2012.

We provided a sample file sampleFile_MIMIC obtained from MIMIC-III (Johnson et al., 2016), a medical dataset of 7,833 newborn patients with 45,674 admissions. This dataset is used for verifying the comorbidity difference between patients with and without PDA based on the *dxpr* package. In this example, we defined PDA cases as patients who had at least one PDA diagnosis (ICD-9-CM 7470*). The controls are defined as patients who never had PDA diagnosis.

### Performance analysis

The *dxpr* package is designated to accelerate the process of large EHR data integration and provide the ready-for-analysis dataset from the integrated EHR data. We verified the running time 100 times with a simulated dataset of 953,294 unique patients and 7,948,418 distinct diagnosis records in a standard personal computer with 64 GB DDR4 2133 GHz RAM and an Intel^®^ Core™ i7-6700 (CPU @3.40 GHz), using Windows 10 (1809), R 4.0.1 (64 bits), and RStudio 1.2.5033.

## Result

### A use case—patients with PDA

**Figure 2:**
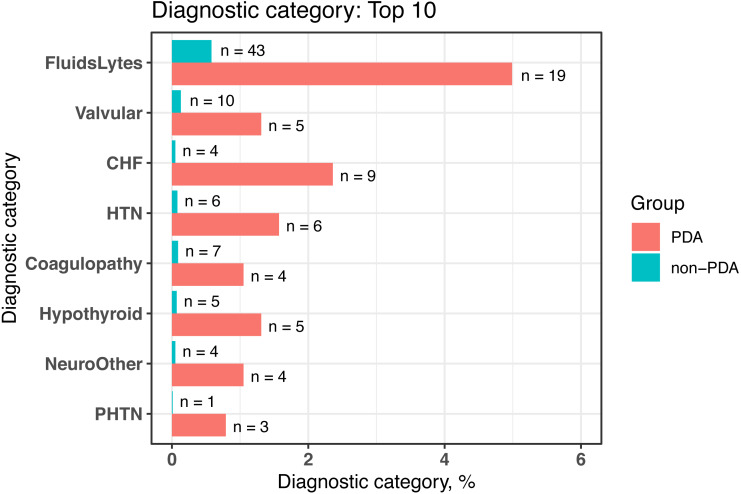
Bar chart to visualize the statistically significant difference of diagnostic categories between patients with and without PDA, grouped by the AHRQ-defined comorbidities. PDA, patent ductus arteriosus; AHRQ, Agency for Healthcare Research and Quality; FluidsLytes, Fluid and electrolyte disorders; Valvular: valvular disease; CHF, congestive heart failure; HTN, hypertension, uncomplicated; Hypothyroid: hypothyroidism; NeuroOther, other neurological disorders; PHTN, pulmonary circulation disorders.

We conducted comorbidity analyses based on a cohort of newborns from MIMIC-III (*n* = 7, 833) by using *dxpr* and tableone (Yoshida & Bartel, 2020) packages. In the *dxpr* package, we first use selectCases function to define case (PDA) and control (non-PDA) groups. In total, 381 and 7,452 patients with and without PAD were included in our study, respectively. Then, icdDxToComorbid function was applied to group diagnoses into AHRQ-defined comorbidities. Finally, we analyzed and graphed the AHRQ-defined comorbidities based on plot_groupedData function (Fig. 2) by using the chi-square test and Fisher’s exact test. To focus on comorbidities that were essential and recorded in adequate individuals in our study population, we excluded comorbidities recorded in <1% of the patients in the PDA or non-PDA group. The analysis-ready data generated by groupedDataLongToWide can be passed to the tableone (Yoshida & Bartel, 2020) package to create objects summarizing all comorbidities stratified by patients with and without PDA and by performing the statistical chi-square tests. The AHRQ comorbidity table revealed 8 of the 16 statistically significant comorbidities (*p* < 0.05, Table 4) among patients with and without PDA, and the comorbidities are visualized in Fig. 2.

**Table 4:** Summary of AHRQ-defined comorbidities based on the tableone package using the integrated data generated by the *dxpr* package.

**AHRQ**^a^**Comorbidities**	**Non-PDA**	**PDA**^b^	**p**
*n*	7452	381	
Coagulopathy (%)	7 (0.1)	4 (1.0)	<0.001
Congestive heart failure (%)	4 (0.1)	9 (2.4)	<0.001
Deficiency anemias (%)	2 (0.0)	1 (0.3)	0.342
Depression (%)	1 (0.0)	0 (0.0)	1
Diabetes, complicated (%)	2 (0.0)	0 (0.0)	1
Fluid and electrolye disorders (%)	43 (0.6)	19 (5.0)	<0.001
Hypertension, complicated (%)	2 (0.0)	0 (0.0)	1
Hypertension, uncomplicated (%)	6 (0.1)	6 (1.6)	<0.001
Hypothyroidism (%)	5 (0.1)	5 (1.3)	<0.001
Other neurological disorders (%)	4 (0.1)	4 (1.0)	<0.001
Peripheral vascular disorders (%)	1 (0.0)	0 (0.0)	1
Pulmonary circulation disorders (%)	1 (0.0)	3 (0.8)	<0.001
Renal failure (%)	1 (0.0)	0 (0.0)	1
Solid tumor without metastasis (%)	1 (0.0)	0 (0.0)	1
Valvular disease (%)	10 (0.1)	5 (1.3)	<0.001
Weight loss (%)	2 (0.0)	0 (0.0)	1

**Notes.**

a AHRQ: Agency for Healthcare Research and Quality.

b PDA: patent ductus arteriosus.

### Performance

For a simulated dataset of 953,294 unique patients and 7,948,418 admission records, code grouping with CCS-defined comorbidities required 149 ± 2.48 s (including code transformation). Case selection required 238 ± 3.05 s to query patients with diseases of the urinary system, eligible period identification required 1.12 ± 0.22 s to find the first and last admission date for each patient, data splitting with the first admission date for each patient required 6.50 ± 0.42 s, condition era generation required 372 ± 6.39 s, and analysis-ready data generation required 3.75 ± 0.27 s.

## Discussion and Conclusions

The *dxpr* package considerably simplifies the extraction, accelerates the processing of clinical data research, and enables researchers to prepare analysis-ready data with a standard workflow. The package had been developed and tested using structured clinical data, such as critical care data (MIMIC-III (Johnson et al., 2016)), a multi-institutional medical care database (Chang Gung Research Database (Tsai et al., 2017; Tseng et al., 2020)), and claims data (National Health Insurance Research Database (Hsieh et al., 2019)), indicating that the package can be applied to data from different countries, institutions, and data structures. The available functions are summarized in Table 5.

**Table 5:** Functions in the *dxpr* package.

**Functions**	**Descriptions**
**I. Data integration**	
icdDxShortToDecimal	Transform ICD^a^ diagnostic codes into decimal format
icdDxDecimalToShort	Transform ICD diagnostic codes into short format
icdDxToCCS	Group ICD diagnostic codes into single CCS^b^ category
icdDxToCCSLvl	Group ICD diagnostic codes into multiple CCS category
icdDxToComorbid	Group ICD diagnostic codes into comorbidity category (Elixhauser, Charlson, and AHRQ)
icdDxToPheWAS	Group ICD diagnostic codes into PheWAS^c^ category
icdDxToCustom	Group ICD diagnostic codes into customized grouping category based on precise method
icdDxToCustomGrep	Group ICD diagnostic codes into customized grouping category based on fuzzy method
**II. Data Wrangling**	
selectCases	Query matching cases in the EHR^d^ data
splitDataByDate	Query data by a clinical event
patientRecordDate	Query the earliest/latest admission date for each patient.
getConditionEra	Calculate condition era by grouped categories of each patient.
groupedDataLongToWide	Convert long format of grouped data into wide format for analytical and plotting functions
**III. Visualization**	
plotICDError	Pareto chart of error ICD list
plotDiagCat	Bar chart of diagnostic categories
**Procedure**	
icdPrToCCS	Group ICD procedure codes into single CCS category
icdPrToCCSLvl	Group ICD procedure codes into multiple CCS category
icdPrToProcedureClass	Group ICD procedure codes into procedure class category

**Notes.**

a ICD, International Classification of Diseases.

b CCS, Clinical Classifications Software.

c PheWAS, Phenome Wide Association Studies.

d EHR, Electronic Health Record.

Several software and packages were developed to facilitate clinical data analysis. rEHR (Springate et al., 2017) established a clinical data analysis workflow to simplify the processing of EHR. The rEHR package simplifies the process of extracting data from EHR databases. It used the database backend that can accelerate data access and process times. However, this design needs database backend, which might not be suitable in many circumstances. Furthermore, the international diagnosis coding standard, such as ICD, were not used in the package. The ICD (Wasey & Lang, 2020) package is designed for calculating comorbidities and medical risk scores with ICD-9 and ICD-10 codes. It is helpful to group ICD codes according to comorbidities. However, in clinical data analysis, eligible case selection, data split based on the defined index date, and visualization are also essential. Therefore, we designed and developed the *dxpr* package to facilitate diagnosis data analysis.

The proposed package has limitations, which come from either the data or package itself. For analyzing clinical data, the *dxpr* package highly depends on diagnosis and procedure codes, but these codes may vary in accuracy across different institutions. Furthermore, the effect of switching diagnosis codes from ICD-9 to ICD-10 should be considered if the analysis period is across the switching date. In addition to diagnosis and procedure data, the other data not included in proposed packages, such as medication data, are important in clinical data analysis projects. In the R ecosystem, the AdhereR (Dima & Dediu, 2017) package implements a set of functions that are consistent with current adherence guidelines and definitions. Fourth, we provide an easy-to-use package that will help analysts process raw data and notify them when potential coding errors exist. However, even with this package, analysts should understand their data precisely. This easy-to-use package will help analysts process clinical data with its coding error–checking functions, but may also lead naïve analysts to miss opportunities to find other errors in the data. Finally, the *dxpr* package is focused on analysis-ready data generation so that the statistic method incorporation may be insufficient. However, the R ecosystem’s most significant advantage is that many well-developed packages were developed to facilitate statistical analysis. In the use case demonstration, our package can be used with other packages, such as tableone package. The tableone (Yoshida & Bartel, 2020) package is developed to ease the construction of the common “Table 1” in research papers, providing patient baseline characteristics table with summary statistics and hypothesis tests.

We demonstrated that the *dxpr* package can play an essential role in complex clinical data preprocessing and analysis-ready data generation through integrating the international standard of clinical data. This package helps clinical data analysts combat the common bottleneck caused by certain clinical data characteristics, such as heterogeneity and sparseness.

## Additional Information and Declarations

### Data Availability

The following information was supplied regarding data availability: The sample code of the dxpr package is available at GitHub:
https://github.com/DHLab-TSENG/dxpr-paper/blob/main/SampleCode.md
A detailed example of the usage of the dxpr package is available at GitHub:
https://github.com/DHLab-TSENG/dxpr-paper/blob/main/Supplement.md
The source code of the dxpr package is available at GitHub:
https://github.com/DHLab-TSENG/dxpr.

## References

[ref-1] Chen G, Khan N, Walker R, Quan H (2010). Validating ICD coding algorithms for diabetes mellitus from administrative data. Diabetes Research and Clinical Practice.

[ref-2] Denny JC, Ritchie MD, Basford MA, Pulley JM, Bastarache L, Brown-Gentry K, Wang D, Masys DR, Roden DM, Crawford DC (2010). PheWAS: demonstrating the feasibility of a phenome-wide scan to discover gene-disease associations. Bioinformatics.

[ref-3] Dima AL, Dediu D (2017). Computation of adherence to medication and visualization of medication histories in R with AdhereR: towards transparent and reproducible use of electronic healthcare data. PLOS ONE.

[ref-4] Elixhauser A, Steiner C, Harris DR, Coffey RM (1998). Comorbidity measures for use with administrative data. Medical Care.

[ref-5] Fleurence RL, Curtis LH, Califf RM, Platt R, Selby JV, Brown JS (2014). Launching PCORnet, a national patient-centered clinical research network. Journal of the American Medical Informatics Association.

[ref-6] Healthcare Cost and Utilization Project (HCUP) (2016). HCUP Procedure Classes. https://www.hcup-us.ahrq.gov/toolssoftware/procedure/procedure.jsp.

[ref-7] Healthcare Cost and Utilization Project (HCUP) (2017). https://www.hcup-us.ahrq.gov/toolssoftware/ccs/ccs.jsp.

[ref-8] Healthcare Cost and Utilization Project (HCUP) (2019a). Beta Elixhauser Comorbidity Software for ICD-10-CM. https://www.hcup-us.ahrq.gov/toolssoftware/comorbidityicd10/comorbidity_icd10.jsp.

[ref-9] Healthcare Cost and Utilization Project (HCUP) (2019b). Beta Clinical Classifications Software (CCS) for ICD-10-CM/PCS. https://www.hcup-us.ahrq.gov/toolssoftware/ccs10/ccs10.jsp.

[ref-10] Hersh WR (2007). Adding value to the electronic health record through secondary use of data for quality assurance, research, and surveillance. American Journal of Managed Care.

[ref-11] Hester J, Wickham H (2021). https://cran.r-project.org/package=odbc.

[ref-12] Hripcsak G, Albers DJ (2013). Next-generation phenotyping of electronic health records. Journal of the American Medical Informatics Association.

[ref-13] Hsieh C-Y, Su C-C, Shao S-C, Sung S-F, Lin S-J, Yang Kao Y-H, Lai EC-C (2019). Taiwan’s National Health Insurance Research Database: past and future. Clinical Epidemiology.

[ref-14] Jensen PB, Jensen LJ, Brunak S (2012). Mining electronic health records: towards better research applications and clinical care. Nature Reviews Genetics.

[ref-15] Johnson AEW, Pollard TJ, Shen L, Lehman LH, Feng M, Ghassemi M, Moody B, Szolovits P, Anthony Celi L, Mark RG (2016). MIMIC-III, a freely accessible critical care database. Scientific Data.

[ref-16] Menendez ME, Neuhaus V, Van Dijk CN, Ring D (2014). The Elixhauser comorbidity method outperforms the Charlson index in predicting inpatient death after orthopaedic surgery. Clinical Orthopaedics and Related Research.

[ref-17] Miotto R, Weng C (2015). Case-based reasoning using electronic health records efficiently identifies eligible patients for clinical trials. Journal of the American Medical Informatics Association.

[ref-18] Moore BJ, White S, Washington R, Coenen N, Elixhauser A (2017). Identifying increased risk of readmission and in-hospital mortality using hospital administrative data. Medical Care.

[ref-19] Observational Health Data Sciences and Informatics (2020). https://www.ohdsi.org/data-standardization/the-common-data-model/.

[ref-20] PCORnet (2020). https://pcornet.org/data-driven-common-model/.

[ref-21] Wickham H, Müller K, R Special Interest Group on Databases (R-SIG-DB) (2021). https://cran.r-project.org/package=DBI.

[ref-22] Raghupathi W, Raghupathi V (2014). Big data analytics in healthcare: promise and potential. Health Information Science and Systems.

[ref-23] Reisinger SJ, Ryan P, O’Hara DJ, Powell GE, Painter JL, Pattishall EN, Morris JA (2010). Development and evaluation of a common data model enabling active drug safety surveillance using disparate healthcare databases. Journal of the American Medical Informatics Association.

[ref-24] Springate DA, Parisi R, Olier I, Reeves D, Kontopantelis E (2017). rEHR: An R package for manipulating and analysing electronic health record data. PLOS ONE.

[ref-25] Tsai MS, Lin MH, Lee CP, Yang YH, Chen WC, Chang GH, Te Tsai Y, Chen PC, Tsai YH (2017). Chang Gung Research Database: a multi-institutional database consisting of original medical records. Biomedical Journal.

[ref-26] Tseng Y-J, Cami A, Goldmann DA, DeMaria A, Mandl KD (2015). Incidence and patterns of extended-course antibiotic therapy in patients evaluated for lyme disease. Clinical Infectious Diseases.

[ref-27] Tseng Y-J, DeMaria A, Goldmann DA, Mandl KD (2017). Claims-based diagnostic patterns of patients evaluated for lyme disease and given extended antibiotic therapy. Vector-Borne and Zoonotic Diseases.

[ref-28] Tseng Y-J, Hu R-F, Lee S-T, Lin Y-L, Hsu C-L, Lin S-W, Liou C-W, Lee J-D, Peng T-I, Lee T-H (2020). Risk factors associated with outcomes of recombinant tissue plasminogen activator therapy in patients with acute ischemic stroke. International Journal of Environmental Research and Public Health.

[ref-29] US Centers for Medicare & Medicaid Services (2017b). ICD-10. https://www.cms.gov/Medicare/Coding/ICD10/index.html.

[ref-30] US Centers for Medicare & Medicaid Services (2017a). ICD-10. https://www.cms.gov/Medicare/Coding/ICD9ProviderDiagnosticCodes/codes.html.

[ref-31] Wasey JO, Lang M (2020). https://cran.r-project.org/web/packages/icd/index.html.

[ref-32] Weiskopf NG, Weng C (2013). Methods and dimensions of electronic health record data quality assessment: enabling reuse for clinical research. Journal of the American Medical Informatics Association.

[ref-33] Wickham H, Hester J, Chang W (2020). devtools: Tools to Make Developing R Packages Easier. https://cran.r-project.org/package=devtools.

[ref-34] Wu J, Roy J, Stewart WF (2010). Prediction modeling using EHR data: challenges, strategies, and a comparison of machine learning approaches. Medical Care.

[ref-35] Yoshida K, Bartel A (2020). https://cran.rproject.org/package=tableone.

